# Analytical validation of a semi-automated methodology for quantitative measurement of SARS-CoV-2 RNA in wastewater collected in northern New England

**DOI:** 10.1128/spectrum.01122-23

**Published:** 2024-05-15

**Authors:** Ashlee A. Robbins, Torrey L. Gallagher, Diana M. Toledo, K. Chase Hershberger, Sabrina M. Salmela, Rachael E. Barney, Zbigniew M. Szczepiorkowski, Gregory J. Tsongalis, Isabella W. Martin, Jacqueline A. Hubbard, Joel A. Lefferts

**Affiliations:** 1 Department of Pathology and Laboratory Medicine, Dartmouth-Hitchcock Medical Center, Geisel School of Medicine at Dartmouth, Hanover, New Hampshire, USA; 2 The Broad Institute at MIT and Harvard, Cambridge, Massachusetts, USA; National Institute of Allergy and Infectious Diseases, Baltimore, Maryland, USA

**Keywords:** COVID-19, sewage, wastewater, RT-ddPCR, RT-qPCR, accuracy, precision

## Abstract

Wastewater**-**based epidemiology (WBE) can be used to monitor the community presence of infectious disease pathogens of public health concern such as the severe acute respiratory syndrome coronavirus 2 (SARS-CoV-2). Viral nucleic acid has been detected in the stool of SARS-CoV-2-infected individuals. Asymptomatic SARS-CoV-2 infections make community monitoring difficult without extensive and continuous population screening. In this study, we validated a procedure that includes manual pre-processing, automated SARS-CoV-2 RNA extraction and detection workflows using both reverse-transcriptase quantitative polymerase chain reaction (RT-qPCR) and reverse transcriptase droplet digital PCR (RT-ddPCR). Genomic RNA and calibration materials were used to create known concentrations of viral material to determine the linearity, accuracy, and precision of the wastewater extraction and SARS-CoV-2 RNA detection. Both RT-qPCR and RT-ddPCR perform similarly in all the validation experiments, with a limit of detection of 50 copies/mL. A wastewater sample from a care facility with a known outbreak was assessed for viral content in replicate, and we showed consistent results across both assays. Finally, in a 2-week survey of two New Hampshire cities, we assessed the suitability of our methods for daily surveillance. This paper describes the technical validation of a molecular assay that can be used for long-term monitoring of SARS-CoV-2 in wastewater as a potential tool for community surveillance to assist with public health efforts.

**IMPORTANCE:**

This paper describes the technical validation of a molecular assay that can be used for the long-term monitoring of SARS-CoV-2 in wastewater as a potential tool for community surveillance to assist with public health efforts.

## INTRODUCTION

Wastewater-based epidemiology (WBE) monitors real-time data about the content of certain biomarkers or chemicals of public health importance in wastewater. Wastewater monitoring has been established in many regions as a tool to detect pathogens and/or community-wide use of chemicals (1–3). A particularly important use of WBE can be found in the detection of poliovirus from asymptomatic community members, enabling targeting of vaccination efforts (4, 5).

The coronavirus disease 2019 (COVID-19) pandemic presents the possibility of using WBE as a lead indicator of community spread of the SARS-CoV-2 virus (6). Nucleic acids of SARS-CoV-2 have been detected in the stool of 40%–60% of patients with an active infection (7). As individuals infected with SARS-CoV-2 do not always present with symptoms, actual community prevalence is difficult to determine without extensive and continuous population screening. WBE has the potential to capture the collective signature of the entire community served by a given wastewater facility, which may be used over time to monitor viral presence, identify new outbreaks, and enable timely decision-making to mitigate viral transmission within the municipality.

WBE has been used to track the presence of SARS-CoV-2 RNA in wastewater sludge (8) and raw wastewater (9–12). In some cases, it has been shown that increases in viral nucleic acid in wastewater precede increases in clinical cases of viral infection (13, 14). The methods used for each step of sample processing and viral detection in these studies have been varied, and there has been no standardized method in the literature to best extract and detect SARS-CoV-2 (3, 5, 15, 16). In-depth descriptions of the validation process for SARS-CoV-2 wastewater surveillance methods are an important element that should be included in reports of novel methods for community surveillance, but this is often lacking in the literature.

In this study, we develop and perform a detailed analytical validation of a SARS-CoV-2 surveillance method using manual pre-processing, automated SARS-CoV-2 extraction, and detection of SARS-CoV-2 using both reverse-transcriptase quantitative real-time polymerase chain reaction (RT-qPCR) and reverse transcriptase droplet digital polymerase chain reaction (RT-ddPCR) methods. RT-ddPCR has been used for SARS-CoV-2 RNA detection in the clinical setting, and one study showed increased sensitivity in detecting low concentrations of SARS-CoV-2 in patient throat swabs (17). Droplet digital PCR has also been shown to have better resistance to PCR inhibitors, particularly in wastewater (18). In this study, we evaluate the analytical sensitivity, linear range, and precision for both RT-qPCR and RT-ddPCR. In addition, primary influent wastewater was collected from two treatment facilities in New Hampshire during periods of relatively low and high COVID-19 case counts as preliminary data demonstrating the potential utility of our methods for WBE surveillance of SARS-CoV-2 infection. Additional surveillance data from our group have been published previously using the methods described and validated in our current manuscript (19).

## MATERIALS AND METHODS

### Samples and control material

Genomic RNA (gRNA) from SARS-CoV-2, Isolate USA-WA1/2020 (BEI Resources, NR-52285) provided at 5.5 × 10^7^ genomic equivalents (ge) per mL was diluted in nuclease-free water or nucleic acid extractions of presumed negative wastewater to specific concentrations (Table 1) and subjected to PCR-based detection methods. The AccuPlex SARS-CoV-2 Verification Panel (LGC SeraCare) including three concentrations of whole genome SARS-CoV-2 viral-based reference material (3, 4, and 5 log copies/mL) was directly subjected to nucleic acid extraction as described below or spiked into clarified wastewater (with or without polyethylene glycol [PEG] concentration) prior to extraction.

**TABLE 1:** Serial dilutions of gRNA

Fold Dilution	ge/mL	ge/Reaction	Log_10_ ge/Reaction
NEAT	5.5 × 10^7^	275,000	5.45
1:10	5.5 × 10^6^	27,500	4.44
1:100	5.5 × 10^5^	2,750	3.44
1:1,000	5.5 × 10^4^	275.0	2.44
1:10,000	5.5 × 10^3^	27.5	1.44
1:100,000	550	2.75	0.44
1:1,000,000	55	0.275	−0.56
1:10,000,000	5.5	0.0275	−1.56

For 14 consecutive days from August 3rd to August 16th 2020, 24-hour composite samples were collected daily from municipal wastewater treatment facilities in Concord, NH (population: 43,244) and Nashua, NH (population: 88,815). Samples were also collected in January 2021 when case counts in those cities were high. Influent wastewater (prior to any treatment) samples were collected over 24-hour periods using refrigerated composite samplers. Additional “grab” or composite collections from the effluent wastewater of long-term care facilities were collected during periods of either SARS-CoV-2 outbreak or zero case counts as determined by routine screening efforts. The SARS-CoV-2-negative “grab” samples from the long-term care facilities were used for linearity spike-in experiments, and SARS-CoV-2-positive grab samples from this facility were used for precision experiments. Wastewater samples (200–1,000 mL) were transported on ice to the testing laboratory for processing and analysis. Collections during times when state and local COVID-19 case counts were low were used as presumably negative samples for spike-in validation studies.

Active SARS-CoV-2 case counts, defined as the number of individuals testing positive in the prior 14 days, for these two municipalities were collected from data provided publicly by the New Hampshire Division of Public Health Services on the COVID-19 dashboard (https://covid19.nh.gov/).

### Wastewater pre-processing

To remove particulate matter, wastewater samples were first centrifuged at 4,000 × *g* (4°C for 30 minutes in 50 mL tubes), yielding a “clarified” wastewater sample. A volume of 40 mL of each wastewater supernatant (“clarified” sample) was transferred to new 50 mL conical tubes. Polyethylene glycol 8000 and NaCl were added and mixed for a final concentration of 10% and 2.25% wt/vol, respectively. Viral particles in the PEG/clarified wastewater solutions were concentrated by centrifugation at 12,000 × *g* for 2 hours at 4°C using a fixed angle rotor. This method of concentration of SARS-CoV-2 nucleic acids by PEG precipitation has been reported by other groups previously (20, 21). The supernatants from this PEG concentration step were discarded, and the viral pellets were re-suspended in 800 µL of nuclease-free water for extraction.

### Extraction

The Wastewater Large-Volume TNA Capture Kit (Promega, Madison, WI) was used to extract the SARS-CoV-2 RNA from the concentrated wastewater samples. Using a 200 µL sample starting volume, the manufacturer’s wastewater extraction protocol was performed with automated processing on the Microlab STAR liquid handling system (Hamilton, Reno, NV). The 50 µL elution was placed into a 96-well plate, and this elution was used for PCR-based detection.

#### Detection methods

SARS-CoV-2 RNA was detected and quantified using two different RT-PCR-based detection methods.

### Reverse transcriptase quantitative PCR (RT-qPCR)

The SARS-CoV-2 RT-qPCR wastewater detection kit (Promega, Madison, WI) workflow uses a multiplexed RT-qPCR method to detect the N1 and/or N2 nucleocapsid gene targets (FAM-labeled probes) developed by the Centers for Disease Control and Prevention (CDC). Each reaction setup includes two control targets: a process control (CY5-labeled probe) and an internal amplification control (HEX-labeled probe). The process control detects Pepper Mild Mottle Virus (PMMV) RNA, a virus found ubiquitously in wastewater samples. The internal amplification control (IAC) is a 435-basepair product from a synthetic DNA template that is included in every reaction.

The Promega RT-qPCR kit includes a quantification standard dsDNA to create a calibration curve and a positive RNA control that were included in each run. Additionally, a 1:100 dilution of the BEI control material was included on each PCR plate.

The PCR conditions followed the following protocol: 15 minutes at 45°C, 2 minutes at 95°C, 40 cycles of 95°C denaturation for 3 seconds, and 62°C annealing for 30 seconds.

Accepted parameters of each RT-qPCR run were a standard curve with a slope between −3.1 and −3.6, an efficiency value >90%, and an R^2^ value >0.99. Valid internal controls required Cq values between 20 and 25 and PMMV Cq values between 20 and 30 cycles.

### Reverse transcriptase droplet digital PCR

RT-ddPCR procedures followed the manufacturer’s instructions of the Droplet Digital PCR system using One-Step RT-ddPCR Advanced Kit for Probes (Bio-Rad, Hercules, CA). RT-ddPCR mixes were converted to droplets using the Automated Droplet Generator (Bio-Rad). The final 20 µL RT-ddPCR mixtures each consisted of: One-Step RT-ddPCR Advanced Kit for Probes (Bio-Rad, Hercules, CA) at a 1× concentration; 20 U/µL reverse transcriptase; 15 mM dithiothreitol (DTT); 450 nM each of either N1 or N2 forward and reverse primers (CDC EUA); 250 nM each of either N1 or N2 probe (CDC EUA); 450 nM each of PPMV forward and reverse primers; 250 nM PMMV probe; and 5.0 µL of nucleic acid sample.

Droplet partitioned reactions were cycled in a thermal cycler (Bio-Rad, Hercules, CA): 60 minutes at 50°C (reverse transcription step), 10 minutes at 95°C, 40 cycles of 94°C denaturation for 30 seconds, and 55°C annealing for 30 seconds, followed by 10 minutes at 98°C and, then, a hold at 4°C.

The cycled plate was then transferred to the QX200 Droplet Reader (BioRad, Hercules, CA) for analysis of droplets in the FAM and HEX channels. Samples with at least one droplet in the positive quadrants were considered a positive signal. Acceptable droplet counts were above 10,000 droplets per well. Acceptable PMMV copies per well were above 1,000 copies per reaction.

### Statistical analysis and data analysis

Quantification of N1 and N2 RNA (copies/µL in the extracted nucleic acid samples) was determined for each sample by both RT-qPCR and RT-ddPCR using the calibration curve created with a dilution series of purified nucleic acid prepared with each run of the RT-qPCR assay and absolute quantification by RT-ddPCR. For samples that were concentrated and/or extracted, the input and output sample volumes for each step were used to back-calculate the original sample concentration (N1 and N2 copies/mL of wastewater) assuming 100% recovery at each step of the concentration and RNA extraction protocol. The SARS-CoV-2 RNA concentration in wastewater samples tested prospectively (see Table 4) was further analyzed to correct for loss of analyte during sample processing using linearity data (see Fig. 3B and D) as a calibration curve to convert from values assuming 100% recovery to values accounting for viral loss/degradation. For example, an initial value of 20 copies/mL calculated from the N1 RT-qPCR assay was converted to a log cp/mL value of 1.301 and used as the y value in the equation y = 1.334 × −1.543 (Fig. 3B) to yield a value of 2.1319 log copies/mL or 135.5 copies/mL.

Expected and observed (measured) concentrations were compared by linear regression analysis using Excel 2016 (Microsoft) with the LINEST function used to determine the residual sum of squares. Potential limits of detection were estimated by reviewing initial linearity data and reproducible detection of analyte at low concentrations in at least 95% of 20 or more replicates. The lowest concentration tested with at least 95% of replicates being detected is designated as the limit detection of the assay.

## RESULTS

### Linearity of RT-qPCR and RT-ddPCR from genomic RNA

**Fig 1:**
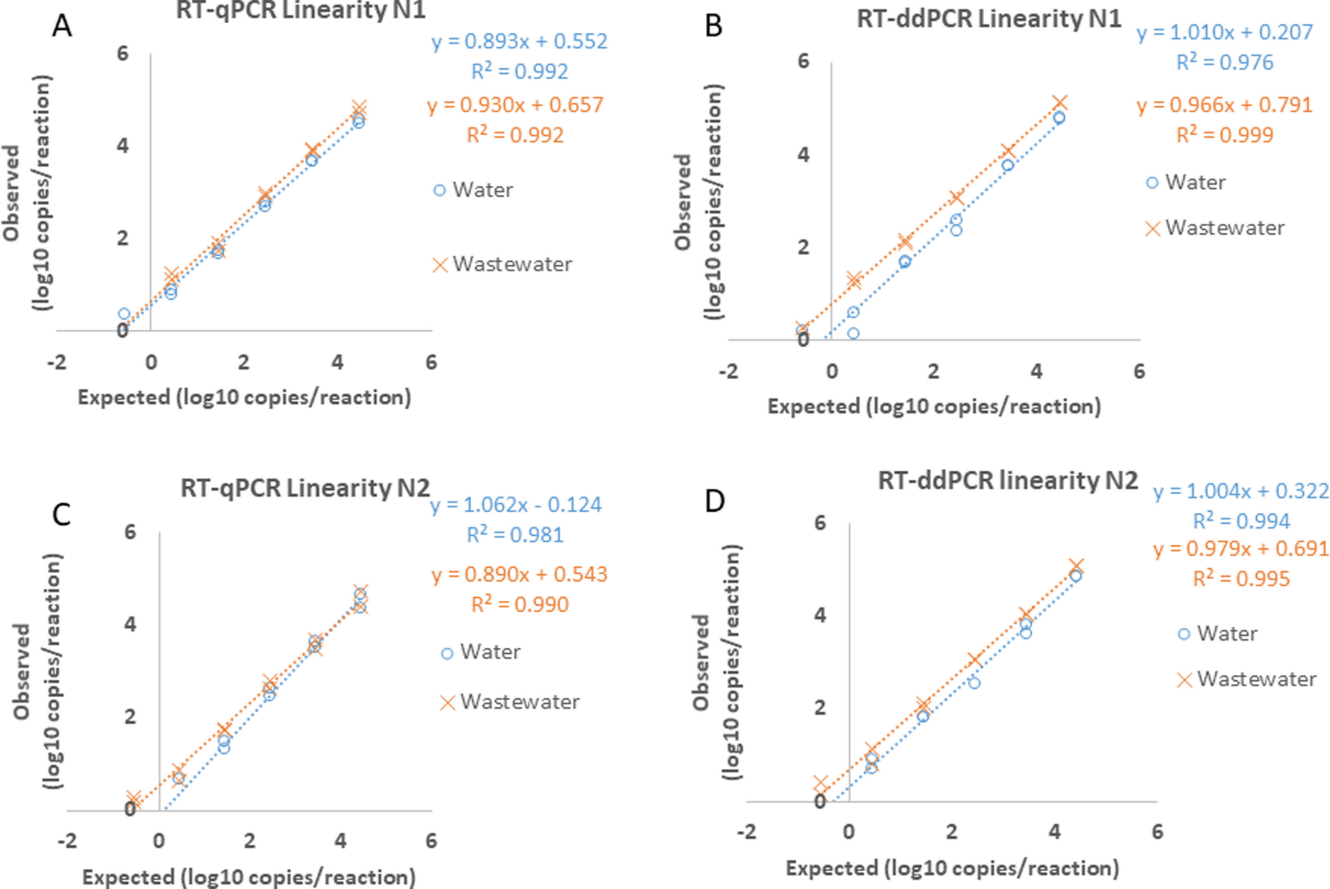
Linearity of gRNA diluted in wastewater or nuclease-free water. A 7-fold serial dilution was created from SARS-CoV-2 gRNA (BEI) diluted in either nuclease-free water (blue circles) or SARS-CoV-2-negative wastewater that had undergone extraction (orange Xs). Expected values converted to log10 (x-axis) were plotted against the measured values of the calculated values converted to log10 (y-axis). Linearity of detection of N1 primers using (**A**) RT-qPCR and (**B**) RT-ddPCR. Linearity of detection of N2 primers using (**C**) RT-qPCR and (**D**) RT-ddPCR. The linear regression equation and coefficient (R^2^) of determination are listed for each graph.

Linearity of both RT-qPCR and RT-ddPCR assays was demonstrated using seven serial dilutions of the BEI (www.beiresources.org) control RNA spanning concentrations of 5.5 × 10^7^ to 5.5 × 10^1^ genomic equivalents (ge)/mL diluted in both nuclease-free water and the resulting elution following nucleic acid extraction of negative wastewater. The concentration values and copies per reaction are shown in Table 1. The linear regression equation and coefficient of determination (R^2^) are listed for each graph of Fig. 1; R^2^ values were greater than 0.99 for all linearity experiments conducted in extracted wastewater and greater than 0.97 for dilutions in nuclease-free water. The resulting residual sum of squares for the RT-qPCR N1 primer was 0.23 and 0.16, and for the N2 primer was 0.37 and 0.34 in nuclease-free water and extracted wastewater, respectively (Fig. 1A and C). The residual sum of squares for the RT-ddPCR N1 primer was 0.69 and 0.04, and the N2 primer was 0.12 and 0.19 for nuclease-free water and extracted wastewater, respectively (Fig. 1B and D). The range of detection for the dilution created in nuclease-free water was 0.275–2.75 × 10^4^ ge/reaction in at least one of two replicates using the N1 CDC primer using both RT-qPCR and RT-ddPCR (Fig. 1A and B). The range of detection was 2.75–2.75 × 10^4^ ge/reaction for the dilution series in nuclease-free water using the N2 CDC primer using both RT-qPCR and RT-ddPCR (Fig. 1C and D). The reportable range for the dilution series in extracted wastewater was 0.275–2.75 × 10^4^ ge/reaction using the N2 CDC primer in both replicates and both detection methods and also for one of the two replicates for the N1 CDC primer using RT-ddPCR (Fig. 1B, C, and D). The dilution series created in wastewater and detected using the N1 CDC primer with RT-qPCR had a range of 2.75–2.75 × 10^4^ ge/reaction (Fig. 1A).

### Accuracy and linearity (extraction and detection methods) of SARS-CoV-2 control samples

**Fig 2:**
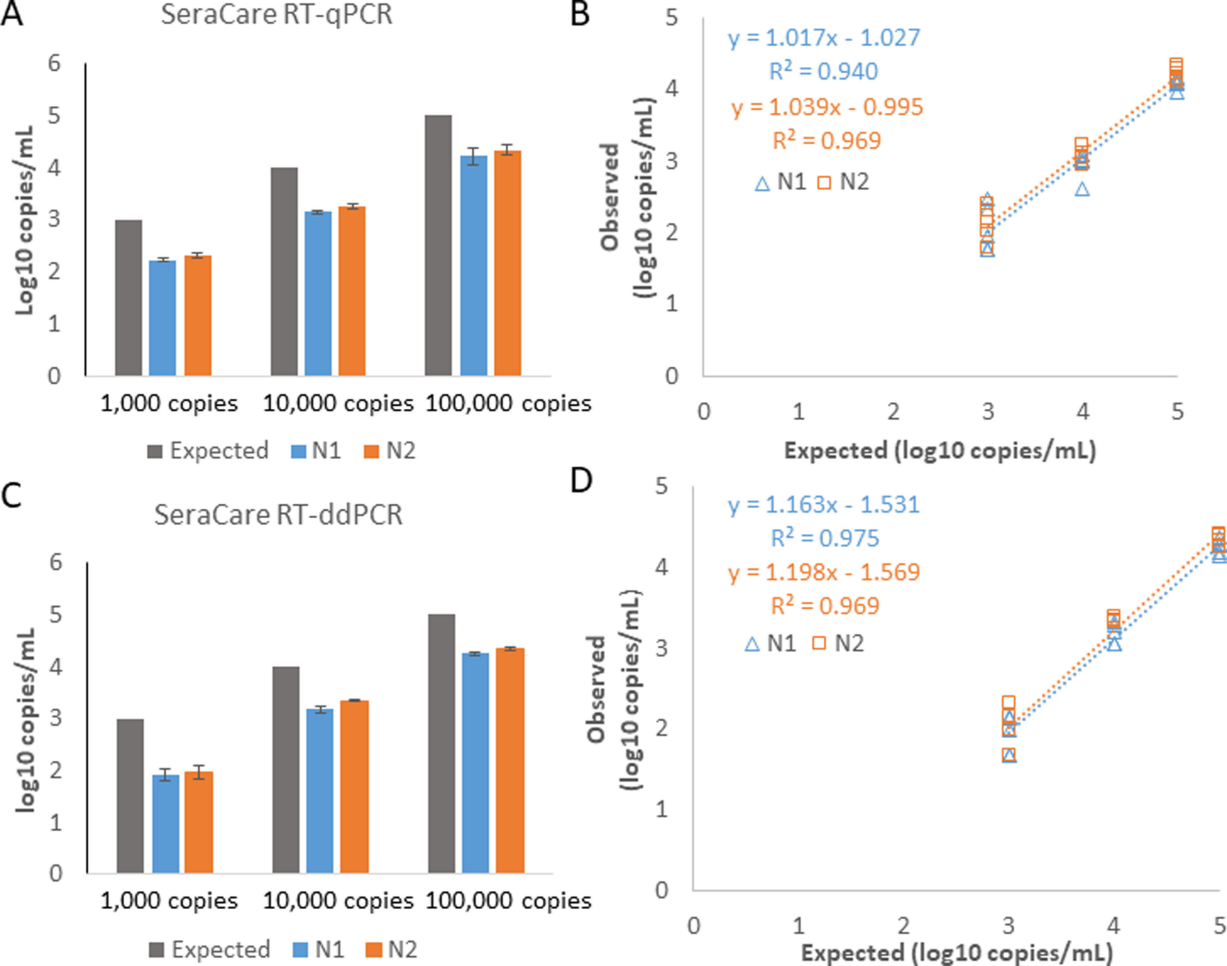
Accuracy of Extracted SeraCare Calibrators. SARS-CoV-2 RNA calibrators were extracted using the Promega wastewater extraction kit. (A and C) Accuracy of replicates of SARS-CoV-2 at three concentrations (copies/mL) by RT-qPCR (A) and RT-ddPCR (C). Measured values were detected between 65% and 85% of the expected values. (B and D) Linearity of three concentrations of SARS-CoV-2 calibrators (1,000 copies/mL, 10,000 copies/mL, and 100,000 copies/mL) detected using RT-qPCR (B) and RT-ddPCR (D). Expected values converted to log10 (copies/mL) were plotted on the x-axis versus the measured values of the calculated concentration converted to log10 (copies/mL). The linear regression equation and coefficient (R^2^) of determination are listed for N1 and N2.

Linearity studies were completed by extracting the three LGC SeraCare verification panel samples in five replicates using the Promega extraction kit. Log-based concentrations of the detected values were calculated and compared with the expected values of the concentration of the starting solution. Detected values were all less than the expected value based on the concentration of the standard materials. For the RT-qPCR assay, the N1 primer had an average log difference of 0.79 log copies/mL, and the N2 primer had an average log difference of 0.69 log copies/mL. The detected values using the RT-ddPCR assay were 0.88 log copies/mL less than expected for N1 and 0.78 log copies/mL less for N2 (Fig. 2A and C). Linearity of the calibration standards is shown in Fig. 2B and D. The residual sum of squares of the RT-qPCR N1 and N2 primers were 0.61 and 0.31, respectively. The residual sum of squares for the RT-ddPCR for the N1 and N2 primers was 0.25 and 0.31, respectively. The RT-qPCR detection method had linearity equations with slopes between 1.01 and 1.04, and all R^2^ values were greater than 0.93. The RT-ddPCR detection method had linearity equations with slopes between 1.16 and 1.2 with R^2^ values greater than 0.96.

### Accuracy and linearity of SARS-CoV-2 detection in spiked clarified wastewater (with and without PEG concentration)

**Fig 3:**
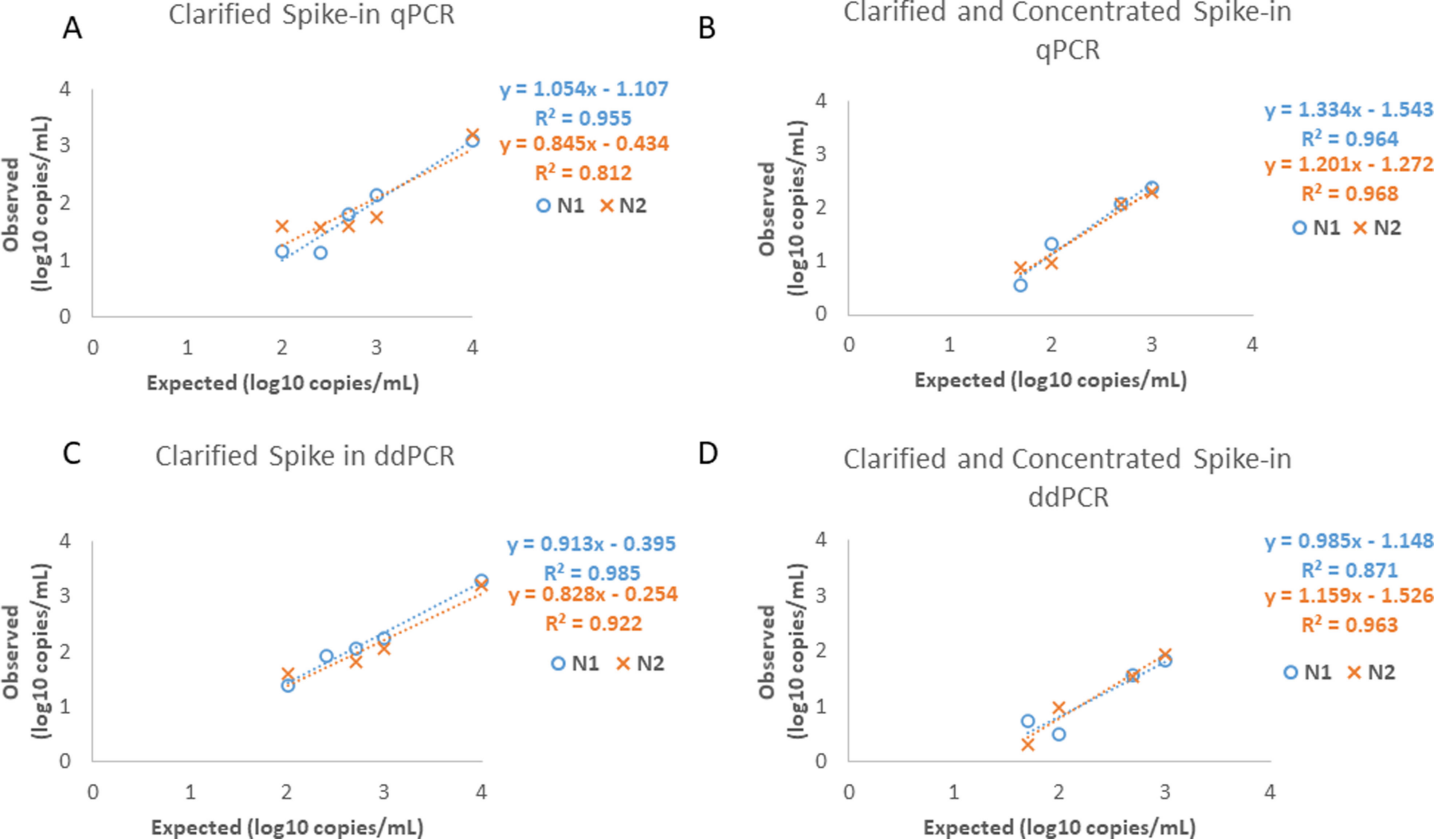
Linearity of contrived samples in wastewater. SeraCare SARS-CoV-2 solutions were spiked into clarified samples and extracted (A, C) or concentrated using the PEG concentration method and then extracted (B, D) Log10-based linearity of expected values (x-axis) versus mean measured values (y-axis) are shown from duplicate experiments. (A, C) SeraCare solution with SARS-CoV-2 concentration of 100,000 copies/mL was spiked into clarified negative wastewater at six concentrations (50, 100, 250, 500, 1,000, and 10,000 copies/mL). (B, D) SeraCare solution was spiked into 20 mL of negative wastewater at four concentrations (50, 100, 500, and 1,000 copies/mL).

The linearity of the extraction and detection methods (without the initial PEG concentration step) was determined by spiking the highest concentration of the LGC SeraCare verification panel into clarified wastewater including dilutions of 50, 100, 250, 500, 1,000, and 10,000 copies/mL. The spike-in samples were then extracted in duplicate. The reportable range for the clarified dilution series was 10^2^–10^4^ copies/mL for both RT-qPCR and RT-ddPCR. The 50 copies/mL dilution was not detected in either assay using either primer set (Fig. 3). The linear regression analysis of the RT-qPCR detection resulted in R^2^ values of 0.9548 and 0.8118 for the N1 and N2 primers, respectively (Fig. 3A). The residual sum of squares for the clarified samples was 0.10 and 0.43 for the N1 and N2 primers, respectively. The average log difference between the detected value and the expected detection for the RT-qPCR detection was 0.954 log cp/mL for N1 and 0.884 log copies/mL for N2. The linear regression analysis of the RT-ddPCR detection resulted in R^2^ values of 0.9854 and 0.9223 for N1 and N2 primers (Fig. 3C). The residual sum of squares for the RT-ddPCR was 0.03 and 0.16 for the N1 and N2 primers, respectively. The average log difference between the detected value and the expected value for the RT-ddPCR detection was 0.642 log copies/mL for N1 and 0.76 log copies/mL for N2.

A second set of clarified wastewater samples spiked to concentrations of 25, 50, 100, 500, and 1,000 copies/mL were processed through the complete assay (including PEG concentration) and subsequently extracted in duplicate (Fig. 3B and D). The clarified and concentrated samples were detected in the dilutions that were tested, and the resultant reportable range was 50–10^3^ copies/mL for both RT-qPCR and RT-ddPCR; the 25 copies/mL dilution was not detected in any of the assays (Fig. 3B and D). The linear regression analysis of the RT-qPCR detection resulted in R^2^ values of 0.9644 and 0.9675 for the N1 and N2 primers, respectively (Fig. 3B). The residual sum of squares of the RT-qPCR detection was 0.43 and 0.04 for N1 and N2, respectively. The average log difference between the detected value and the expected value for the RT-qPCR detection was 0.76 log copies/mL for N1 and 0.79 log copies/mL for N2. The linear regression analysis of the RT-ddPCR detection resulted in R^2^ values of 0.8709 and 0.9626 for N1 and N2 primers (Fig. 3D). The residual sum of squares of the RT-ddPCR was 0.14 and 0.04 for the N1 and N2 primers. The average log difference between the detected value and the expected value for RT-ddPCR detection was 1.18 log copies/mL for N1 and 1.15 log copies/mL for N2. In addition to demonstrating linearity of our assay, the linear regression equations shown (Fig. 3B and D) are also used below to correct wastewater SARS-CoV-2 values that assume 100% analyte recovery.

When comparing the concentrated versus clarified-only samples, we found that the detectable limit is lower in the concentrated samples with detection at 50 copies/mL, whereas the clarified-only samples have a lower limit of 100 copies/mL. The RT-qPCR R^2^ values were higher for both N1 and N2 when the samples underwent PEG concentration. Additionally, the mean difference between the expected value and detected values was lower for the concentrated samples in the RT-qPCR assay. The RT-ddPCR R^2^ value was lower for PEG-concentrated samples when using the N1 primer, but higher for the PEG-concentrated samples when using the N2 primer. Using the RT-ddPCR assay, the concentrated samples had a higher mean difference between the expected values and the detected values in the concentrated samples.

### Limit of detection (spike prior to PEG concentration, extraction, and detection)

Wastewater was spiked using the SeraCare calibration material to create 24 samples with a concentration of 50 copies/mL, and 24 samples of 100 copies/mL each. These concentrations were selected due to their reliability of detection in the range of detection studies described previously. The samples were concentrated through the PEG protocol, extracted and subjected to RT-qPCR and RT-ddPCR. The rates of positive detection are shown in Table 2. The 50 copies/mL and 100 copies/mL samples were detected with either the N1 or N2 primers in 95.8% of samples with RT-qPCR, and 100% of samples with RT-ddPCR. When examining performance of the N1 and N2 assays separately, the N1 assay detected 75% of samples with RT-qPCR and 87.5% of samples with RT-ddPCR at 50 copies/mL. The detection rates for the N2 assay were 66.7% for RT-qPCR and 91.7% for RT-ddPCR at 50 copies/mL. At 100 copies/mL, N1 was detected in 91.7% of samples using RT-ddPCR and 95.8% of samples using RT-qPCR; N2 was detected in 95.8% of samples using RT-ddPCR and 87.5% of samples using RT-qPCR (Table 2). The observed limit of detection was 50 copies/mL or lower, established for both detection methods since the N1 and/or N2 targets were detected in at least 95% of replicates with a concentration of 50 copies/mL.

**TABLE 2:** Detection rate of N1 and N2 targets in RT-qPCR and RT-ddPCR for low concentration precision samples

	RT-qPCR		RT-ddPCR	
	50 copies/mL	100 copies/mL	50 copies/mL	100 copies/mL
N1	18/24 (75%)	23/24 (95.8%)	21/24 (87.5%)	22/24 (91.7%)
N2	16/24 (66.7%)	21/24 (87.5%)	22/24 (91.7%)	23/24 (95.8%)
N1 or N2	23/24 (95.8%)	23/24 (95.8%)	24/24 (100%)	24/24 (100%)

**Fig 4:**
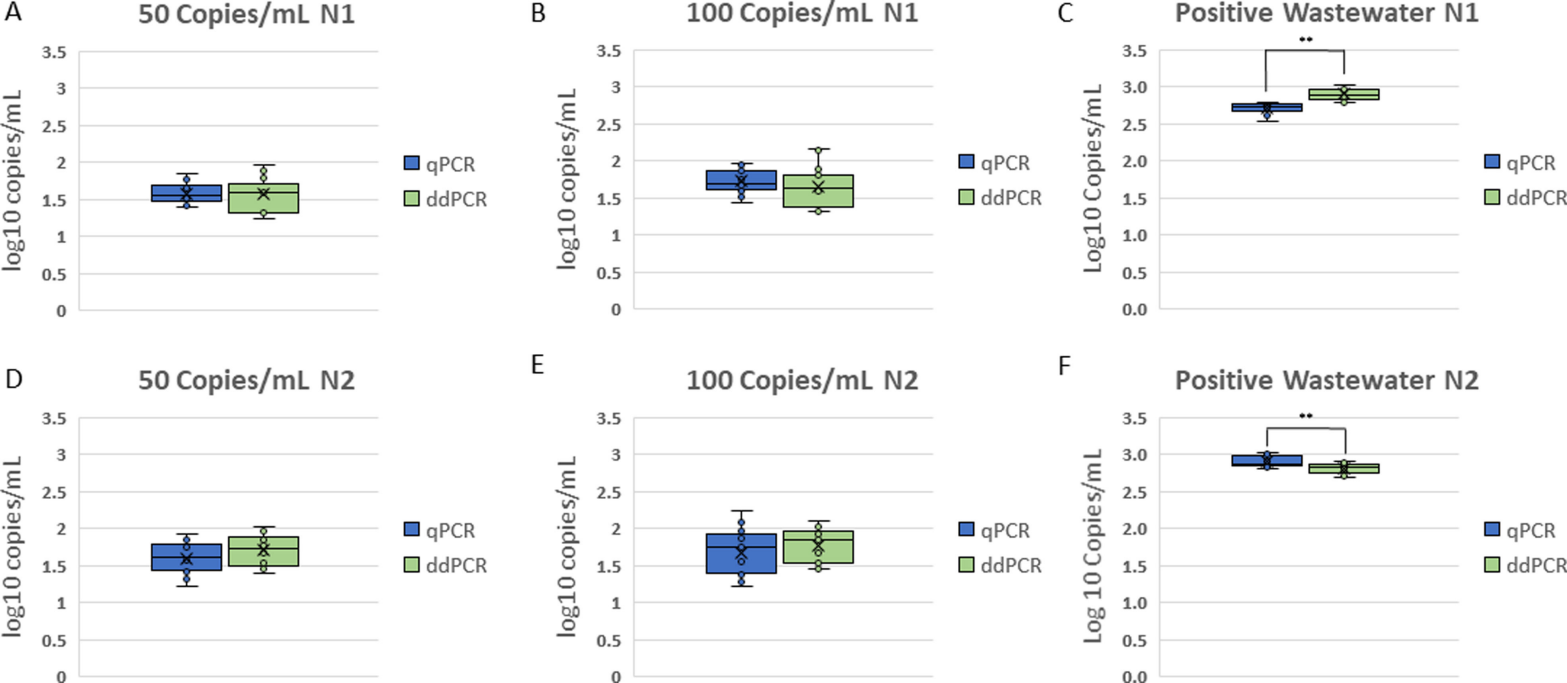
Precision of wastewater extractions. SeraCare SARS-CoV-2 solutions were spiked into clarified wastewater and were concentrated along with a wastewater sample from a region with a known cluster of active cases of COVID-19. Detected concentrations (log10 copies/mL) for both the SeraCare spiked and positive wastewater samples are shown for N1 (**A, B, C**) and N2 (**D, E, F**) using qPCR (blue) and ddPCR (green). When comparing between qPCR and ddPCR for each primer, the N1 and N2 primers are significantly different (*P* < 0.005).

The initial concentration of the samples with detectable SARS-CoV-2 RNA was 50 copies/mL, and the standard curve of the RT-qPCR resulted in an average concentration calculated as 39.4 ± 2.97 copies/mL and 42.72 ± 5.43 copies/mL using the N1 primer for RT-qPCR and RT-ddPCR, respectively (Fig. 4A). Using the N2 primer, the concentrations were 44.23 ± 5.09 copies/mL and 63.34 ± 8.45 copies/mL for RT-qPCR and RT-ddPCR (Fig. 4D). The calculated concentration of the samples with an initial concentration of 100 copies/mL using the N1 primer was 55.37 ± 4.02 and 51.81 ± 7.34 copies/mL for RT-qPCR and RT-ddPCR, respectively (Fig. 4B), and using the N2 primer, the concentrations were 58.01 ± 8.56 and 65.44 ± 6.25 copies/mL for RT-qPCR and RT-ddPCR (Fig. 4E). There were no significant differences between the detection levels of RT-qPCR and RT-ddPCR for spiked samples with 50 copies/mL or 100 copies/mL tested with either primer-probe set (Student’s *t*-test).

### Precision of wastewater detection

Samples of known positive wastewater were collected from a long-term care facility during a COVID-19 outbreak. Replicate 40 mL samples (*n* = 8) were concentrated and extracted in duplicate and then analyzed by RT-qPCR and RT-ddPCR. The average calculated concentration based on the detection of the N1 target was 2.71 ± 0.01 log copies/mL (%CV = 2.42) for RT-qPCR and 2.89 ± 0.02 log copies/mL (%CV = 2.78) for RT-ddPCR; the N2 concentrations were 2.90 ± 0.02 log copies/mL (%CV = 2.46) for RT-qPCR and 2.81 ± 0.01 log copies/mL (%CV = 2.47) for RT-ddPCR (Fig. 4C and F). A student’s *t*-test of the concentrations between the N1 and N2 detection is significantly different for RT-qPCR (*P* = 5.4 x 10^−9^) and RT-ddPCR (*P* = 0.004). When comparing RT-qPCR and RT-ddPCR detection, a significant difference was noted for N1 (*P* = 4.46 x 10^−8^) and N2 (*P* = 0.001) (Table 3).

**TABLE 3:** Precision SARS-CoV-2 RNA measurements of positive wastewater (*n* = 8)

	RT-qPCR	RT-ddPCR
Mean cp/mL (%CV) N1	513.37 (13.97)	796.74 (18.51)
Mean cp/mL (%CV) N2	806.48 (16.81)	655.57 (15.48)
Mean log cp/mL (%CV) N1	2.48 (2.64)	2.89 (2.78)
Mean log cp/mL (%CV) N2	2.65 (2.68)	2.81 (2.47)

### Detected SARS-CoV-2 in municipal wastewater

Daily sampling of primary influent from wastewater treatment facilities in Concord and Nashua, New Hampshire were tested for SARS-CoV-2 RNA during August 2020. In Concord, the reported active case counts during the study period were less than four total cases each day (Table 4). There were 3 days when SARS-CoV-2 RNA was detected in the municipal wastewater samples. Two of these instances were detected by RT-ddPCR only, and one was detected by RT-qPCR only. In Nashua, the active case counts ranged from 27 to 52 cases on any given day during the study period. The wastewater tested positive on 9 of 14 days: 7 of the 9 days detected with RT-ddPCR and 4 of the 9 days detected with RT-qPCR. Concurrent detection by both assays occurred in samples on 2 days as shown in Table 4. Additional samples collected in January of 2021 when the case counts were much higher in both cities have higher concentrations of SARS-CoV-2 and show consistent detection in both assays with both sets of primers. Concentrations calculated with the assumption that 100% of the viral material was recovered are shown first, and viral concentrations corrected for analyte lost during the various sample processing steps (PEG concentration and nucleic acid extraction) are shown second (bold and in parentheses). Samples with concentrations above our assay’s 50 copies/mL limit of detection (LOD) (corrected values) are detected more consistently with both N1 and N2 targets in both assays (Table 4).

**TABLE 4:** Detection of SARS-CoV-2 RNA in serial wastewater collections from two wastewater treatment facilities in New Hampshire^*a*^

	City 1: Concord, NH; Population: 43,412					City 2: Nashua, NH, Population: 89,246				
	RT-qPCR N1	RT-qPCR N2	RT-ddPCR N1	RT-ddPCR N2	Cases	RT-qPCR N1	RT-qPCR N2	RT-ddPCR N1	RT-ddPCR N2	Cases
8/3/2020	TND	TND	TND	1.2 (**24.3**)	<4	TND	1.5 (**16.1**)	TND	1.4 (**27.7**)	49
8/4/2020	TND	TND	TND	TND	<4	3.1 (**33.5**)	TND	1.2 (**17.6**)	TND	52
8/5/2020	TND	TND	TND	TND	<4	TND	TND	TND	1.2 (**24.3**)	52
8/6/2020	TND	TND	1.2 (**17.6**)	TND	<4	TND	TND	TND	7.2 (**113.9**)	52
8/7/2020	TND	TND	TND	TND	<4	TND	TND	TND	TND	46
8/8/2020	TND	TND	TND	TND	<4	TND	TND	TND	TND	41
8/9/2020	TND	TND	TND	TND	<4	TND	TND	TND	TND	34
8/10/2020	TND	TND	TND	TND	<4	TND	1.3 (**14.3**)	TND	TND	33
8/11/2020	TND	TND	TND	TND	<4	TND	TND	1.4 (**20.6**)	TND	35
8/12/2020	TND	TND	TND	TND	<4	2.6 (**29.4**)	TND	TND	TND	27
8/13/2020	TND	TND	TND	TND	<4	TND	TND	1.4 (**20.6**)	TND	32
8/14/2020	TND	TND	TND	TND	<4	TND	TND	TND	TND	40
8/15/2020	TND	TND	TND	TND	<4	TND	TND	TND	TND	36
8/16/2020	TND	1.6 (**16.9**)	TND	TND	<4	TND	TND	1.4 (**20.6**)	TND	28
1/13/2021	42.9 (**240.1**)	17.6 (**124.8**)	48 (**745.3**)	48 (**585.1**)	230	20 (**135.5**)	18.2 (**128.3**)	24 (**368.7**)	24 (**321.7**)	536
1/20/2021	15.3 (**110.9**)	6.7 (**55.8**)	22 (**337.6**)	38 (**478.3**)	210	34.2 (**202.6**)	11.5 (**87.6**)	30 (**462.5**)	24 (**321**.7)	523

^*a*^ Samples were collected daily for 14 days from August 3rd to August 16th 2020. Measured values (copies/mL) from qPCR and ddPCR from the same extracted sample are displayed based on an assumption of 100% recovery and also as corrected copies/mL values that follow in parentheses (bolded) taking into account intrinsic loss of viral material during processing steps. Corrected values are calculated by applying equations from Figure 3B and 3D. TND = ‘target not detected’. Cases are the number of individuals testing positive in the prior 14 days per the New Hampshire Division of Public Health Services dashboard. Bottom two rows: two wastewater samples collected during a period when COVID-19 case counts were high in January of 2021.

## DISCUSSION

Previous studies have investigated whether SARS-CoV-2 RNA can be reliably detected in wastewater, but a standardized method has yet to be established (1, 13–16, 20–24). Studies assessing reproducibility in interlaboratory studies have shown comparable results with multiple variations in pre-treatment methods as well as concentration methods (25, 26). We set out to validate a SARS-CoV-2 detection method in a Clinical Laboratory Improvement Amendments (CLIA)-licensed , College of American Pathologists (CAP)-accredited laboratory setting and highlight key assay performance characteristics we believe should be addressed in publications describing any wastewater SARS-CoV-2 detection method (Table 5).

**TABLE 5:** Summary of select assay performance characteristics

Assay Parameter	RT-qPCR (N1)	RT-qPCR (N2)	RT-ddPCR (N1)	RT-ddPCR (N2)
RNA detection only				
Reportable range/linearity: gRNA (ge/reaction); Fig. 1	0.275–2.75 × 10^4^	2.75–2.75 × 10^4^	0.275–2.75 × 10^4^	2.75–2.75 × 10^4^
Reportable range/linearity: gRNA in negative wastewater extraction (ge/reaction); Fig. 1	2.75–2.75 × 10^4^	0.275–2.75 × 10^4^	0.275–2.75 × 10^4^	0.275–2.75 × 10^4^
RNA extraction/detection				
Mean difference (expected-measured values) SeraCare calibrators; Fig. 2A and C	0.79 log cp/mL	0.69 log cp/mL	0.88 log cp/mL	0.78 log cp/mL
Mean difference (expected-measured values) SeraCare Calibrators spiked in clarified wastewater; Fig. 3A and C	0.95 log cp/mL	0.88 log cp/mL	0.64 log cp/mL	0.76 log cp/mL
PEG concentration, RNA extraction/detection				
Mean difference (expected-measured values) SeraCare calibrators in PEG concentrated wastewater; Fig. 3B and D	0.76 log cp/mL	0.79 log cp/mL	1.18 log cp/mL	1.15 log cp/mL
LOD (N1 and/or N2): SeraCare Calibrators in PEG concentrated wastewater; Table 2	50 cp/mL (95.8% detection)		50 copies/mL (100% detection)	
Precision testing of known positive wastewater (*n* = 8): mean value (%CV); Fig. 4	2.48 log cp/mL (2.64%CV)	2.65 log cp/mL (2.68%CV)	2.89 log cp/mL (2.78%CV)	2.81 log cp/mL (2.47%CV)

The first set of experiments that we describe determines the linearity of the RT-qPCR and RT-ddPCR assays using SARS-CoV-2 genomic RNA (BEI). The results show that SARS-CoV-2 RNA spiked into extracted wastewater has a similar detectable range and linearity as SARS-CoV-2 RNA spiked into nuclease-free water. Subsequent experiments validate a commercial wastewater extraction kit (Promega, Madison, WI) using the SeraCare calibration materials with detection by RT-qPCR and RT-ddPCR. In addition, we looked at the amount of viral recovery from the automated extraction of the three concentrations of SeraCare calibration materials. Finally, we used the full protocol to determine SARS-CoV-2 positivity in wastewater collected as 24-hour composite samples from two municipality wastewater treatment facilities.

Our data established that we could detect SARS-CoV-2 at concentrations of 50 cp/mL or lower in wastewater using a starting sample volume of 40 mL. The lower limit of detection and precision are comparable between RT-qPCR and RT-ddPCR, with reliable detection at starting spike-in concentrations of 50 copies/mL that undergo PEG concentration. When compared with the range of theoretical LODs of between 3.0 and 6.1 log GC/L reported in the interlaboratory method evaluation by Pecson et al, our LOD of 50 copies/mL (50,000 copies/L) falls within that range at 4.7 log copies/L (25). The method of detection is important to consider when investigating the viral load in wastewater of small rural towns in areas of low prevalence where viral loads may be low or only sporadically positive.

In our analysis of 24-hour composite wastewater samples from Nashua and Concord, NH, we saw inconsistent levels of positivity occurring over the 2-week study collection period. This sporadic detection is likely due to the low infection rate in these communities during that time. Additionally, loss and degradation of viral material during collection, transport, and storage may have contributed to the low-level detection and lack of detection. Since the infection rates were low in those communities during that time, the amount of SARS-CoV-2 genetic material present in the wastewater could have been near or below the limit of detection of our assay, resulting in a lack of detection or inconsistent detection in these August 2020 samples. In addition, weather events such as rain can change the relative concentration of virus and viral nucleic acids in the wastewater stream. The two samples collected in January 2021, when the number of infected individuals in those cities was significantly higher, both show positivity in both N1 and N2 assays for the RT-qPCR and RT-ddPCR as shown in Table 4.

Each processing step from collection at the wastewater facility through extraction of nucleic acids may be associated with a certain amount of loss of the original contents of the sample. In a collected wastewater sample, our first processing step was clarification, which is useful for removing large, interfering solids but may result in loss of SARS-CoV-2 signal, since some of the genetic material can be bound to the solids in the wastewater (27, 28). Another factor to consider is that the 40 mL samples were clarified and then stored at −80°C before further processing. This freeze-thaw cycle can contribute to the degradation of the RNA in solution and result in lower detection rates. The concentration step can have some additional loss if all the nucleic acids in the sample are not precipitated and captured. Our measured viral concentrations at the LOD, using both RT-ddPCR and RT-qPCR, are ~10% of the concentration of the original sample in our spike-in studies. This 10% recovery suggests a significant loss of viral material during the PEG concentration and extraction steps, which is similar to what has been reported in previous studies (27, 29). Our analysis of actual wastewater samples includes measurements that do not account for this intrinsic loss of viral target and additional measurements that correct for the loss observed during the PEG concentration and nucleic acid extraction steps. This dual analysis highlights the challenges in comparing results between studies using different methods of testing and analysis. Given these potential limitations, it is difficult to determine the exact amount of virus in the original wastewater samples that we collected from participating sites. We therefore strongly recommend the introduction of calibration material into the assay as early in sample processing as possible.

The processes used by investigators to concentrate SARS-CoV-2 nucleic acids in wastewater are highly variable in the literature. Our study uses a concentration method that dissolves PEG 8000 and sodium chloride into clarified wastewater to concentrate nucleic acids; these two products are commonly used chemicals that are readily available in most research laboratories. PEG concentration has been shown to be an effective way of concentrating enveloped viruses (20, 25, 27, 29). During the pandemic, when supply chain issues posed major problems for researchers, this method provided a simple option for concentrating nucleic acids in wastewater without having to rely on access to filters that are used for ultracentrifugation.

Looking at the presence of SARS-CoV-2 in wastewater at one time point provides limited information to make important decisions regarding public health. Tracking changes or trends in SARS-CoV-2 RNA concentration in wastewater over serial collections would be a better indicator of changes in infection prevalence within a population and would be more useful in making changes in public responses. Compared with previous efforts to monitor wastewater for poliovirus as confirmation of eradication, SARS-CoV-2 will unlikely be eradicated in the near future. Accurate and sensitive quantitative wastewater measurements and not just qualitative wastewater surveillance will be more useful for population SARS-CoV-2 monitoring, since low levels of the virus will likely be present in most wastewater globally. Our WBE approach as described in detail in the current study has been applied to a separate surveillance study that correlates measured concentrations of SARS-CoV-2 RNA in wastewater with community case counts (19).

In this current study, we present a detailed account of our analytical validation of a workflow for the detection and quantification including additional suggestions for testing and data analysis of SARS-CoV-2 RNA in wastewater. We stress the need for accurate quantitative methods achieved through thorough validation and methods that account for sample processing, nucleic acid extraction, and detection. Although we are unable to directly compare our results with those of other laboratories, the recent introduction of quantitative International Units for SARS-CoV-2 (30) and interlaboratory programs to compare results between different test systems can lead to improved standardization.
